# Operando Raman Gradient Analysis for Temperature-Dependent Electrolyte Characterization

**DOI:** 10.1021/acsenergylett.4c00954

**Published:** 2024-07-03

**Authors:** Lorenz F. Olbrich, Ben Jagger, Johannes Ihli, Mauro Pasta

**Affiliations:** Department of Materials, University of Oxford, Oxford OX1 3PH, U.K.

## Abstract

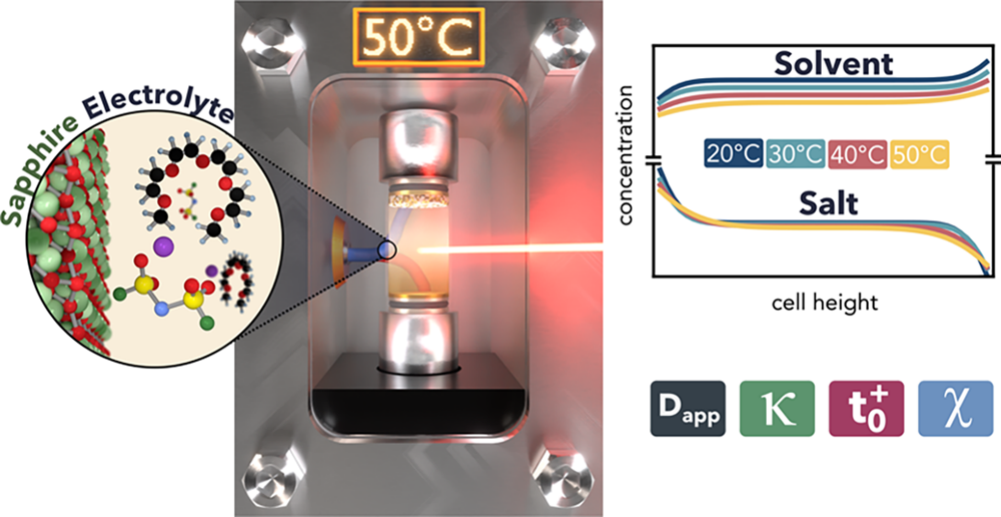

Transport and thermodynamic properties are integral parameters to understand, model, and optimize state-of-the-art and next-generation battery electrolytes. The accurate measurement of these properties is experimentally challenging as well as time- and resource-intensive, and consequently, reports are scarce. Their dependence on temperature is explored even less and is commonly limited to a few temperature points. Recently, we introduced an operando Raman gradient analysis (ORGA) tool to extract transport and thermodynamic properties. Here, we expand the capabilities of ORGA by incorporating a temperature-sensitive external reference into the design. With this enhancement, we are able to visualize the local concentration of any Raman-active species in the electrolyte and detect lithium filament nucleation. We demonstrate and validate this new functionality of ORGA via an examination of lithium bis(fluorosulfonyl)imide (LiFSI) in tetraethylene glycol dimethyl ether (G4) as a function of temperature. All transport properties and activation energies are reported, and the effect of temperature is discussed.

Transport and thermodynamic properties of an electrolyte are crucial determinants of battery rate capability. Properties, such as the Fickian (or “apparent”) diffusion coefficient (*D*_app_), the ionic conductivity (κ), the lithium transference number (), and the molar thermodynamic factor (χ_M_), influence the uniformity of lithium plating, filament nucleation, and the overall cycle life.^1,2^ The temperature dependence of these properties is rarely measured in the literature despite cell temperatures changing significantly during operation, especially during fast charging and/or discharging, where temperatures can reach up to 80 °C.^3,4^ Conventional methods for measuring electrolyte properties encompass restricted diffusion measurements,^5,6^ high-frequency electrochemical impedance spectroscopy (EIS),^7−9^ the Hittorf method,^6,10,11^ and the assessment of liquid junction potentials in concentration cells,^6,10^ among others. However, these conventional methods are resource-intensive and have various drawbacks.^12,13^

An alternative approach involves calculating *D*_app_ and  by measuring salt concentration as a function of position and time during polarization and fitting time-resolved concentration gradients to equations from concentrated solution theory (CST).^5,12^ In recent years, several operando characterization techniques have been developed to visualize these concentration gradients, including magnetic resonance imaging (MRI),^14,15^ nuclear magnetic resonance (NMR) imaging,^16,17^ and laser interferometry.^18^

We recently extended and refined this approach by combining electrochemical measurements with operando confocal Raman microspectroscopy in a technique we called operando Raman gradient analysis (ORGA).^12,13,19^ In addition to gradient-extracted *D*_app_ and , chronopotentiometry (CP) and electrochemical impedance spectrocopy (EIS) allow for the calculation of κ from the resistance observed in high-frequency EIS and χ_M_ from the concentration overpotential η_s_ and the interfacial gradient , enabling the determination of all four electrolyte properties in a unified setup and in a single measurement.^12^

A fundamental limitation of concentration gradient techniques, including ORGA, is the need for a suitable reference to account for laser energy fluctuations and to enable comparability across multiple measurements. So far, a vibrational mode of the solvent has been used to normalize all spectra, which limits the visualization of gradients to the solute.^12,13,19^

Here we introduce an external reference, extending ORGA’s capabilities to the visualization of concentration gradients of any Raman-active species in the electrolyte, including the solvent. We first validate this novel approach by visualizing the concentration gradients of both salt and solvent, using 1*m* lithium bis(fluorosulfonyl)imide (LiFSI) in tetraethylene glycol dimethyl ether (G4) as a model system. We then calculate and compare the transport and thermodynamic properties obtained with this method with the traditional internal reference approach. We then use the setup to investigate the temperature dependence of the transport and thermodynamic properties in the range 20 to 50 °C and discuss their physical meaning. Finally, we demonstrate how the external reference further enables the detection of lithium filament nucleation.

Time-resolved lithium concentration gradient visualization enables the calculation of transport properties as discussed in earlier publications and briefly described in the Supporting Information (SI, Figure S1).^12,13^ This is achieved by quantifying concentration-sensitive vibrational modes of the salt as a function of polarization time along the *z*-axis of a vertically oriented optical cell using Raman microspectroscopy as schematically illustrated in Figure 1a.^12^

**Figure 1:**
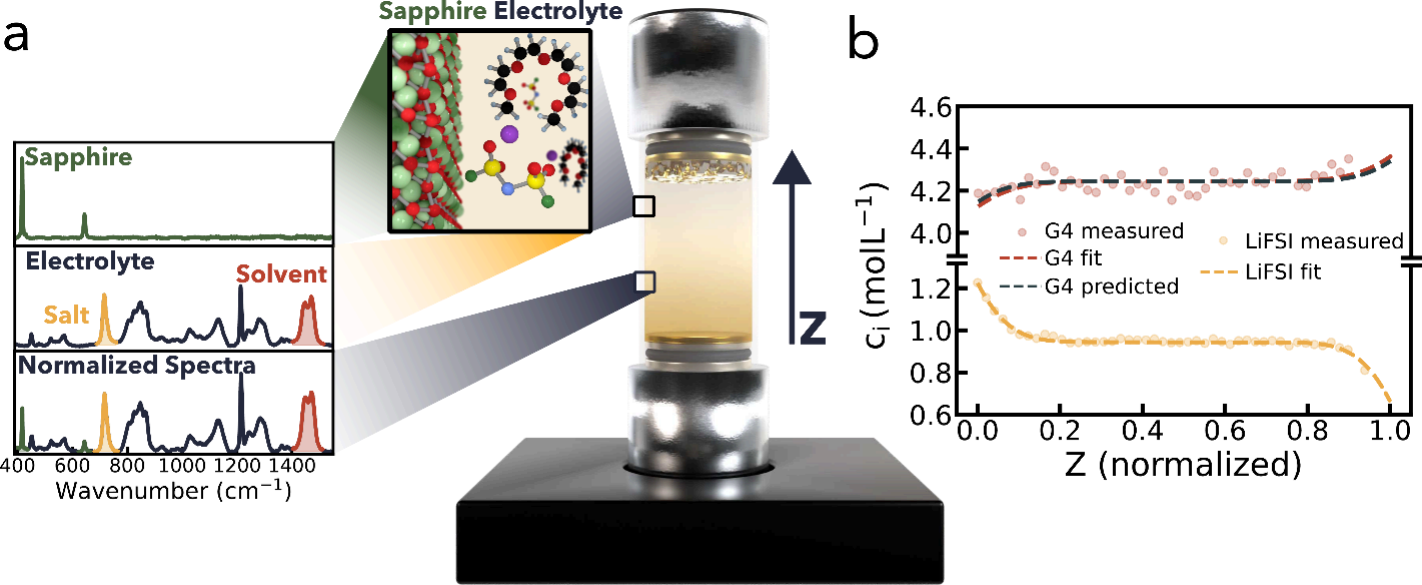
**Operando Raman Gradient Analysis using an External Reference.** (**a**) Schematic of an ORGA cell setup with a field-of-view external reference incorporated into the cell body. The Raman spectra of the isolated cell body (single-crystal sapphire), the isolated electrolyte (1 *m* LiFSI G4), and the normalized spectra measured in the ORGA setup where reference and electrolyte overlap are shown. (**b**) Using the external reference to normalize the spectra enables visualization of all Raman-active species in the electrolyte, including solute and solvent. The experimentally measured LiFSI and G4 concentrations are plotted as a function of normalized cell height. The LiFSI and G4 concentrations are both fit according to CST. The G4 fit is compared to the predicted G4 concentration calculated from the local LiFSI concentration and the corresponding partial molar volumes.

Previous studies utilized a vibrational mode of the solvent as an internal reference to normalize all Raman spectra.^12^ This method hinges on the assumption that interfacial solvent reactions, solvent migration, and Faradaic convection are negligible.^20^ Essentially, it assumes that variations in salt concentration solely determine the local solvent concentration.^20,21^ An alternative approach to address this limitation involves introducing an internal standard within the electrolyte solution. However, such a molecule must meet several criteria: (1) it should not affect the solvation structure of ions in solution, (2) it must be chemically compatible with the electrolyte and electrodes, (3) it should possess active Raman modes with a high extinction coefficient to minimize the required concentration, and (4) it must lack an electric dipole moment to prevent migration under an electric field. Needless to say, identifying such a molecule is highly improbable.

The most rigorous approach is to introduce a field-of-view external reference. Single-crystal sapphire has exceptional chemical resistance, good durability, and compressive strength. Moreover, it exhibits high optical transmittance and distinct, sharp vibrational Raman peaks, as depicted in Figure 1a. We, therefore, developed a cell made of single-crystal sapphire (Figure 1a). When measuring Raman spectra in the sapphire-incorporated ORGA setup, both the electrolyte and sapphire peaks can be detected simultaneously (Figure 1a). By adjusting the laser focal point, their relative intensity can be tuned to a distance where both signal intensities are maximized to reduce noise-induced uncertainty (see Figure S2). Subsequently, all spectra can be normalized using the A_1g_ vibrational mode (418 cm^–1^) of sapphire and thereby correct for laser-induced intensity fluctuations.^22^ This new spectrum normalization method improves measurement accuracy by overcoming the assumptions described earlier and allows monitoring of concentration profiles of any Raman-active species in the electrolyte.

The normalized spectra can now be used to quantify the local lithium concentration. Since lithium does not exhibit any Raman scattering itself, its concentration can be determined by quantifying the anion concentration and, assuming charge neutrality, equating the local FSI^–^ concentration, [FSI^–^], to the concentration of Li^+^, [Li^+^]. This assumption is generally valid, since the Debye screening length of electrolytes is in the nanometer scale, 3 orders of magnitude smaller than the probing volume of the confocal laser.^5^ The FSI^–^ S–N–S bending peak (from 680 to 760 cm^–1^), for example, can be used to quantify [FSI^–^] (Figure S3a). Peak area integration, rather than peak intensity evaluation, yields more accurate [Li^+^] gradients. This is because as the [FSI^–^] increases, the fraction of contact-ion-pairs (CIPs) and aggregates (AGGs) also increases, resulting in peak broadening and formation of secondary “shoulder peaks”.^12^

Figure S3b shows normalized areas as a function of [Li^+^] obtained from a conventional calibration line measurement. A two-point operando calibration line is also plotted (linear fit of *c*_s,ini_ through the origin), assuming a linear area-to-concentration ratio. The operando calibration line shows an excellent fitting with *R*^2^ = 0.9992 and 0.9988 up to 2 M LiFSI and 2.5 M LiFSI, respectively. Since ORGA measurements are conducted in a concentration range of about *c*_s,ini_ ± 0.5 M (here 0.5 to 1.5 M LiFSI in G4), we conclude that the linear operando calibration line can be used to convert normalized areas into concentrations. Commercially relevant electrolytes operate within a similar concentration range and thus are also expected to exhibit a linear area-to-concentration relationship.^23,24^

To verify the validity of the new normalization and calibration method, the concentration gradients of a 1 *m* LiFSI in G4 at 20 °C cell were processed using the conventional (solvent normalization and ex situ calibration curve) and new (sapphire reference and operando calibration) methods. Figure S3c shows two exemplar concentration gradients for a line-scan collected after 2 h of polarization. While most calculated concentrations align with both processing methods, one key difference can be observed close to the plating side (normalized *z* approaching 1). [Li^+^] seems to decrease rapidly when determined by the external reference processing method. A similar behavior is also observed in the G4 concentration gradient (Figure S4). Upon inspection of the ORGA cell after polarization (Figures 2a and S5), a significant amount of lithium dendrites can be observed. When the dendrites grow from the plating electrode into the cell, the amount of electrolyte in the probing volume is reduced, and thus, both *c*_s_ and *c*_0_ decline. Using the conventional normalization, the effect of lithium dendrites is obscured, since both salt and solvent Raman bands decline simultaneously. The addition of an external reference to ORGA thus enables the monitoring of filament nucleation as discussed later.

The transport properties calculated from the concentration gradients (described in detail in ref (12) and in the SI, Figure S6) are listed in Table 1 and show satisfactory agreement.

**Table 1:** Comparison of Transport Properties Calculated from Conventional (Solvent Normalization and Ex Situ Calibration) and New (External Reference Normalization and Operando Calibration) Raman Processing Methods

**Method**	***D***_**app**_**(m**^**2**^ **s**^**–1**^**)**	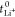	**χ**_**M**_
**Conventional**	(6.3 ± 0.5) × 10^–11^	0.49 ± 0.06	1.72 ± 0.12
**External Reference**	(6.3 ± 0.6) × 10^–11^	0.50 ± 0.06	1.82 ± 0.06

Figure 1b shows the measured concentration gradients of LiFSI and G4, the fitted LiFSI gradient, the fitted G4 gradient, and the predicted G4 gradient, calculated from the local LiFSI concentration and the corresponding partial molar volumes. The measured G4 gradient follows the predicted trend of depletion and accumulation at the stripping and plating sides, respectively. The G4 fit and the predicted G4 values are in good agreement. However, the measured G4 concentrations show a larger measurement noise which originates from small fluctuations of the stage position, as described in the SI (see two-point operando calibration section, Figure S7). Since the G4 concentration (*c*_0,ini_) is about 4 times larger than the LiFSI concentration, the measurement uncertainty is amplified, resulting in an apparent larger measurement noise. To the best of our knowledge, this is the first time both the solute and solvent of an electrolyte are visualized. This paves the way to guide and verify theorists in their ongoing ambitions to advance the theoretical understanding of transport in commercially relevant multicomponent electrolytes.^25^

Besides gradient visualization, the external reference can be used to track the time when lithium dendrites are formed and thus provide an additional functionality to the ORGA setup. Figure 2b shows a heatmap of all the Raman spectra collected in one ORGA run. The external-reference-normalized spectra are integrated and plotted as a function of lithium stripping time and cell height *z*. At the *z*-value closest to the plating side, a gradual intensity loss after around 3 h of lithium stripping can be observed. After around 7 and 9 h, the intensity loss extends further into the ORGA cell. Figure 2c shows the corresponding, normalized Raman spectra as a function of stripping time verifying the electrolyte (all salt and solvent peaks) intensity loss with respect to the external reference peaks (see 410  and 610 cm^–1^). Three additional signal-intensity heat maps are shown in Figure S8. Accordingly, a slow reduction in interphase impedance can be observed with increasing stripping time (Figure S9), consistent with filament nucleation.^26^ This functionality can be used to track and compare lithium filament nucleation in different electrolyte systems and under different measurement conditions as demonstrated later.

**Figure 2:**
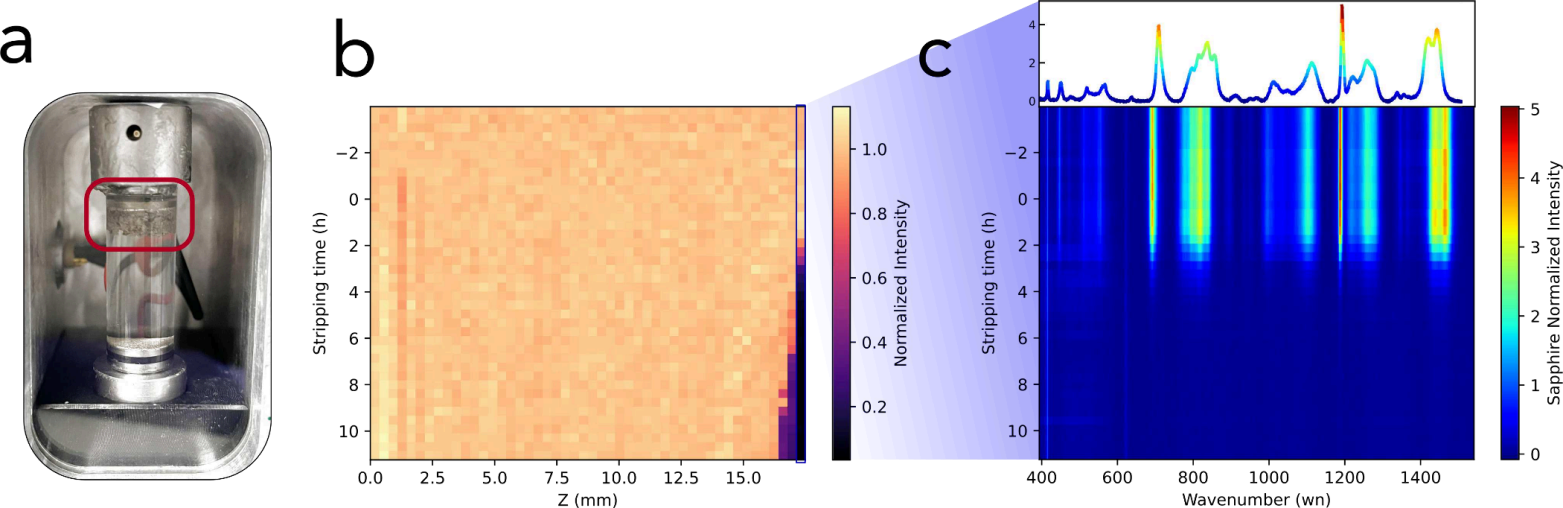
**Filament Nucleation Monitoring**. (**a**) Photograph of a postmortem ORGA cell, which shows how lithium filaments have formed on the lithium plating side. (**b**) Heatmap showing the integrated intensity of each (external reference normalized) Raman spectrum as a function of stripping time (*y*-axis) and cell height (*x*-axis). (**c**) Shows the acquired normalized Raman spectra as a function of stripping time at the highest *z*-value, i.e., closest to the plating side.

To extend the parameter space of measurement conditions, we developed an ORGA-compatible environmental chamber, which is able to regulate the ORGA cell temperature. Measurement and chamber details are provided in the SI (Figures S10–12).

To determine the electrolyte’s temperature during an ORGA experiment, we utilize sapphire’s bond-distance temperature dependency. A material’s Raman-active bond distance and temperature are positively, and linearly, correlated, with peaks shifting to a lower wavenumber as the temperature increases.^27^Figure S13 shows the relative peak shift of the A_1g_ vibrational mode of sapphire as a function of temperature. A linear relationship between temperature increase and peak shift can be observed (linear fit, *R*^2^ = 0.997), validating that the temperature set point and ORGA temperature increase accordingly. The temperature calibration line and its corresponding slope of −0.013 cm^–1^ K^–1^ can be used to determine temperature set points across future experiments.

To cross-validate the peak shift method, the κ determined in ORGA is compared to measurements in a blocking-electrode conductivity cell (Figure 3a). The results are in good agreement thus verifying the electrolyte temperature reading accuracy. An increase in κ from 2.8  to 5.9 mS cm^–1^ is observed between 20 to 50 °C, reflecting the increased charge carrier mobility due to the reduced electrolyte viscosity at elevated temperatures.^23^ Arrhenius fitting shows excellent agreement (*R*^2^ = 0.9997, see Figure 3a inset) with an activation energy of 0.19 eV. The activation energy is more than twice as high as for a 1 *m* LiFSI in 1,2-dimethoxyethane electrolyte (DME) electrolyte reported elsewhere (0.08 eV).^28^ Considering that 1 *m* LiFSI DME exhibits a significantly lower viscosity (1.137 mPa s at 20 °C) compared to its G4 electrolyte analogue (12.30 mPa s at 20 °C), this can at least partially account for the increased temperature sensitivity and, consequently, the higher activation energy observed in κ in LiFSI G4.

**Figure 3:**
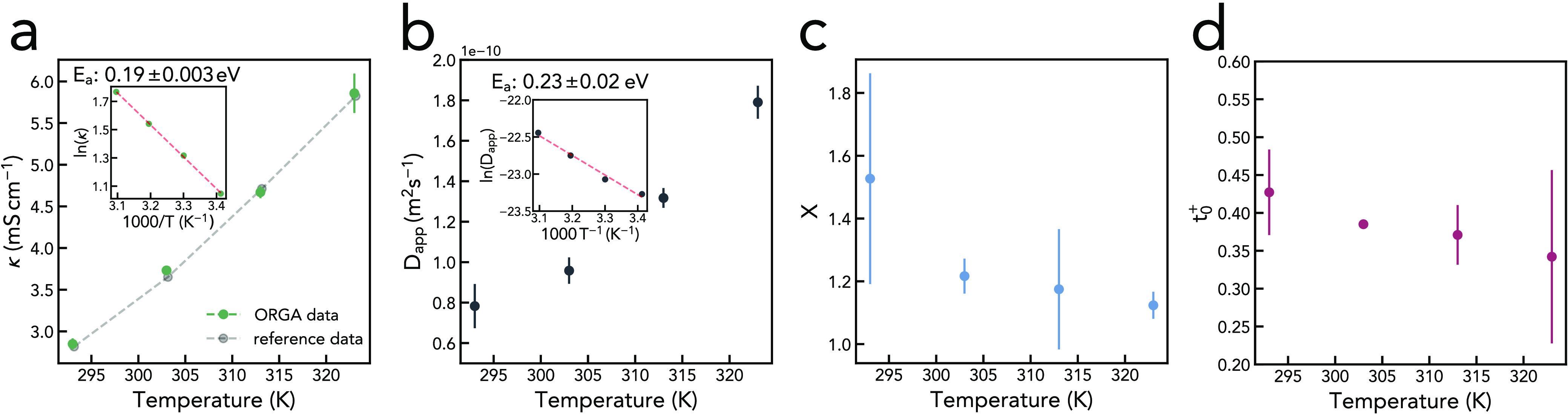
**Temperature Dependence of Electrolyte Transport Properties.** (**a**) Shows κ as a function of temperature obtained through EIS measurements in ORGA and reference data determined via EIS in a conductivity cell. The inset shows an Arrhenius fit. (**b**) Shows *D*_app_ as a function of temperature. The inset shows Arrhenius fit. (**c**) χ_M_ as a function of temperature (**d**)  as a function of temperature.

Figure 3b shows *D*_app_ as a function of temperature. Just like κ, *D*_app_ depends on the ionic mobility, and thus, a positive correlation of *D*_app_ and temperature can be observed with an increase from 0.78 × 10^–10^  to 1.79 × 10^–10^ m^2^ s^–1^ from 20 to 50 °C. However, the increase is more rapid, which is reflected in a larger activation energy of 0.23 eV determined by Arrhenius fitting (see Figure 3b inset, *R*^2^ = 0.9926). While both κ and *D*_app_ strongly depend on the ionic mobility, κ is even more sensitive to the number of free charge carriers than *D*_app_.^23,29^ This could be an indication that the solvation environment of lithium is changing as a function of temperature.

For a better understanding of the solvation environment, χ_M_ is plotted as a function of temperature in Figure 3c. According to the extended Debye–Hückel theory, quantifying the electrostatics of ions in solution, χ_M_, and its variation with concentration and temperature are reflective of the nonideal behavior of electrolytes.^6,30^ Despite the larger error bar, a general decrease of χ_M_ from 1.53 to 1.12 can be observed between 20 and 50 °C in Figure 3c. While there is no universal understanding of how temperature affects χ_M_ at moderate concentrations, short-range species interactions such as ion association and pairing are expected to play a crucial role.^5,10,31,32^ In fact, it has been previously suggested that the increase in χ_M_ with solute concentration could be explained by an increased interaction between the solvent and ions, which causes a decrease in the solvent vapor pressure, resulting in increased salt activity.^10,33^

Reports on temperature dependencies of χ_M_ are scarce due to the laborious nature of conventional measurement techniques, with the existing few reporting varying temperature dependencies, even within the same electrolyte chemistries.^9,29,34^

Figure 3d shows the lithium transference number  as a function of temperature. A slight decrease from 0.42 to 0.34 is clearly visible indicating that less current is transported by Li^+^ cations compared to FSI^–^ at elevated temperatures. Similar to χ_M_, the temperature dependency of  is scarcely reported. The few reports vary in their conclusions, some describing  as independent of temperature,^9,35^ whereas others observe a temperature dependency in either direction.^29,36^ In any case,  and its temperature dependence are expected to depend strongly on electrolyte chemistry and the ion coordination structure.

To complement the discussion on the temperature dependency of χ_M_ and , the coordination structure of FSI^–^ was investigated, focusing on the S–N–S bending peak (Figure 4a) in the Raman spectra. Indeed, the peak shows the growth of a shoulder with temperature, indicative of the formation of more CIPs. As shown in Figure S14, the peaks can be deconvoluted using pseudo-Voigt fitting.^37^ The analysis indicates an increase of [FSI^–^]_bound_/([FSI^–^]_bound_ + [FSI^–^]_free_) from around 50 to around 59% between 20 and 50 °C. This is an interesting finding, since the effect of temperature on ion association is far from trivial and strongly dependent on the investigated electrolyte formulations. Since lithium salt dissociation in organic solvents is generally exothermic, a shift toward more associated ions could be rationalized at higher temperatures.^38^ Besides, the extent to which association dominates is primarily influenced by the relative dielectric constant (ϵ_r_) of the solvent. A smaller ϵ_r_ results in weaker ion solvation and thus increased association and vice versa. Wang et al. recently demonstrated this in a comparative study of LiPF_6_ in propylene carbonate (PC) and ethyl methyl carbonate (EMC).^10^ ϵ_r_ generally decreases with increasing temperature thus favoring ion association.^39^ However, entropic gains through dissociation become energetically more relevant with increasing temperature.^40^ The multitude of competing contributions toward solvation and thus χ_M_ and  at different temperatures highlights the complexity of the concepts, and a definite conclusion is beyond the scope of this publication. Yet, it underlines the importance of being able to experimentally access transport properties at different conditions within a unified setup. All thermodynamic and transport properties, and the corresponding activation energies, determined in this study are listed in Table 2.

**Figure 4:**
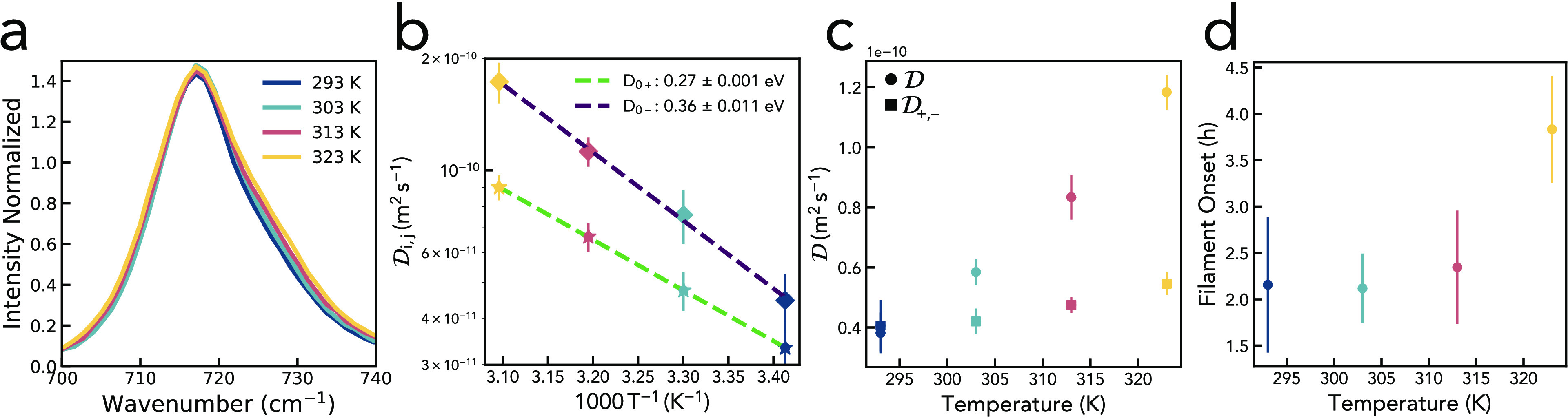
**Temperature Dependence of Electrolyte Transport Properties.** (**a**) S–N–S bending peak of FSI^–^ for different temperatures. (**b**) OSM diffusion coefficient for different temperatures with Arrhenius fit. (**c**) Thermodynamic diffusion coefficient and the  OSM diffusion coefficient. (**d**) Average filament onset time as a function of temperature.

**Table 2:** Transport and Thermodynamic Properties as a Function of Temperature

***T* (°C)**	***D***_**app**_**(m**^**2**^ **s**^**–1**^**)**	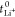	**χ**_**M**_	**κ (mS cm**^**–1**^**)**
**20**	(0.78 ± 0.11) × 10^–10^	0.43 ± 0.06	1.53 ± 0.33	2.85 ± 0.07
**30**	(0.96 ± 0.07) × 10^–10^	0.39 ± 0.01	1.22 ± 0.06	3.73 ± 0.03
**40**	(1.32 ± 0.05) × 10^–10^	0.37 ± 0.04	1.17 ± 0.19	4.67 ± 0.07
**50**	(1.79 ± 0.08) × 10^–10^	0.34 ± 0.11	1.12 ± 0.04	5.86 ± 0.24
***E***_***a***_**(eV)**	0.23 ± 0.03	—	—	0.19 ± 0.003

The increasing amount of CIPs with temperature aligns with the earlier observation that the activation energy of κ is smaller than that of *D*_app_. This is because ionic conduction is strongly affected by the amount of free charge carriers, which is reduced by the formation of CIPs.

Having measured κ, *D*_app_, , χ_M_, and partial molar volumes allows further calculation of the thermodynamic  and Onsager-Stefan-Maxwell (OSM) diffusion coefficients at each temperature point (see SI for calculation). In contrast to *D*_app_, the OSM diffusion coefficients provide a more detailed description of frictional interactions between each species ( = Li^+^–solvent,  = FSI^–^–solvent,  = Li^+^–FSI^–^).  is affected by salt chemical potential gradients rather than concentration gradients and thus considers gradients created by temperature, pressure, or composition differences. It is used for a more comprehensive and fundamental understanding of diffusion processes and is especially relevant in theoretical models and simulations.^41,42^Figure 4b,c shows the temperature dependency of Stefan-Maxwell Diffusion coefficients and . Both  and  increase with temperature from 3.3 × 10^–11^  to 9.0 × 10^–11^ m^2^ s^–1^ and 4.5 × 10^–11^  to 1.7 × 10^–10^ m^2^ s^–1^ respectively. Moreover, they both show excellent agreement with Arrhenius fitting (*R*^2^ > 0.999)  is larger than  at all temperatures, since FSI^−^ has a lower charge density than Li^+^ and thus is less solvated by G4, reducing its drag. This is also reflected in the  < 0.5. As seen in Figure 4b,  has a larger activation energy than ; thus, their differences in diffusion become more pronounced with increasing temperature. This supports the observation that  is reducing with temperature as relatively more charge is carried by the anion. Figure 3c demonstrates that , along with , generally increases with temperature. However, unlike the more pronounced increases observed for , , and , the rise in  with temperature is marginal. As demonstrated before, more CIPs are formed at elevated temperature. Consequently, Li^+^ and FSI^−^ have stronger correlated motion, which negatively contributes to , counteracting the increased ion mobility induced by the increased thermal energy.

Lastly, the filament onset time was calculated based on the intensity loss of normalized Raman spectra as described in Figure 2. Figure 4d shows the average time stamp where the integrated Raman intensity dropped below 75% of its initial value as a function of temperature. The data indicates that with increasing temperature, the filament propagation is delayed. A similar trend has been observed elsewhere.^43^ As shown in Table 2, Li^+^ transport is accelerated at elevated temperatures, and thus, Li^+^ depletion at the plating lithium electrode surface is slowed down, which, according to Sand’s theory,^44^ should delay inhomogeneous plating. However, within the time scale of the ORGA measurements (10 h), the [Li^+^] at the plating electrode should not reach zero, emphasizing the multifaceted origins of lithium filament nucleation and the importance of properties of the lithium-solid electrolyte interphase (SEI).^45^

In conclusion, this paper introduces a single-crystal sapphire cell body as an external reference in the ORGA setup. We validate the reliability of this novel approach by comparing the measured transport and thermodynamic properties of the model electrolyte system LiFSI in G4 to those measured using the traditional approach, which employs the solvent as internal standard. Furthermore, we extend the investigation to explore the temperature-dependence of these properties. As expected, we observe an increase in ionic conductivity and diffusion coefficient, and we report the respective activation energies. In contrast,  and χ_M_ decrease with increasing temperature. We attribute this behavior to the formation of more contact ion pairs as the temperature increases. The temperature-dependence of the OSM diffusion coefficients is in agreement with this hypothesis. We also visualize, for the first time, the concentration gradients of both salt and solvent, paving the way for the investigations of the most relevant electrolyte systems comprising multiple components (salt additives and cosolvents). Finally, we demonstrate the capability to detect lithium filament growth.^12,46^

The addition of an external reference makes ORGA a holistic experimental approach which combines the measurement of temperature-dependent thermodynamic and transport properties with macroscopic cycling phenomena to ultimately help elucidate the origin of dendrite formation and support the down-selection of the most promising Li-metal anode electrolyte formulations.

## Supporting Information Available

Experimental procedures including materials, cell assembly, ORGA operation, data processing and property calculations, partial molar volume and SVF calculations, two-point calibration. Supporting figures (PDF)
