# Supplementary material for: Operando Raman Gradient Analysis for Temperature-Dependent Electrolyte Characterization
Source: ACS Energy Lett. 2024 Jul 3;9(7):3636–42. doi: 10.1021/acsenergylett.4c00954 (PMC11250066; doi:10.1021/acsenergylett.4c00954)
Supplement: Supplementary file 1 — nz4c00954_si_001.pdf [file nz4c00954_si_001.pdf]

Operando Raman Gradient Analysis for Temperature-Dependent Electrolyte Characterization

Lorenz F. Olbrich[1], Ben Jagger[1], Johannes Ihli[1], and Mauro Pasta[1]

[1]Department of Materials, University of Oxford, Oxford OX1 3PH, United Kingdom

Experimental Procedures

Materials

Lithium bis(fluorosulfonyl)imide (LiFSI) (Battery Grade-99%) was purchased from Fluorochem Ltd. Tetraethylene glycol dimethyl ether (G4) (anhydrous, 99%+) was purchased from Sigma Aldrich. Handling of LiFSI and G4 was always performed in an argon-filled glovebox (MBraun) with low H_2O content (<1 ppm) and low O_2 content (<1 ppm). LiFSI was dried further under a high vacuum at 70 °C for 48 h. G4 has dried over 3 Å molecular sieves, which were washed and then dried for one week. All glassware was dried at 80 °C under vacuum before being used and brought into the glovebox. The H_2O content of the electrolyte solutions was determined by Karl Fischer titration, also performed in an argon-filled glovebox, and recorded to be below 15 ppm of H_2O. Experimental information describing density measurements and partial molar volume calculations are described below.

Cell Assembly

Two stainless steel pistons were designed to fit inside a single-crystalline tube with 20 mm in length, with an inner diameter (ID) of 8 mm and an outer diameter (OD) of 10 mm. The sapphire tubes were custom made by Rayotek Scientific, Inc (CA, US). An interelectrode distance of 15 mm was chosen because it was sufficiently large to detect a concentration gradient with good spectral resolution and the diffusion layer not to progress too quickly into the centre of the cell, which would make the fitting equation invalid. If it were longer the diffusion layer would not progress quickly enough, and the measurement would take substantially longer. Each piston was further equipped with an O-ring made of FFKM for increased chemical resistance against ethers. The lithium metal foil (99.9%, 750 µm thickness, Alfa-Aesar) acting as lithium reservoir and electrodes for the symmetric cell, was prepared by scraping off the native oxide layer of the foils and calendaring to a thickness of 300 µm. The ORGA cell was assembled in an Ar-filled glovebox. Circular lithium discs were prepared with a diameter of 8 mm and placed onto each of the stainless steel pistons. One piston was then placed inside the quartz tube, ~1 mL of electrolyte was added, and then the second piston was introduced on the opposite side cell, sealing the cell. As an extra layer of protection from atmospheric contamination the cell was sealed using a UV Curing Optical Adhesive NOA61 from THORLABS Ltd (UK). Care was taken not to introduce any gas bubbles into the system. Before any tests were run, the cell was set up inside the A Renishaw inVia Reflex laser confocal Raman microscope equipped with a near-IR 785 nm laser and connected to a Biologic SP150 potentiostat. Important to note, the cell was vertically placed on the sample stage to avoid natural convection.

ORGA Operation

The cell was rested for four hours prior to drawing a current to enable lithium passivation. 1D line scans in the z-direction (along the cell height) were continuously measured throughout the experiment. A 1200 lines mm^{-1} grating, a 5× magnification objective (Leica, 0.12 NA, 14 mm WD) providing a 7.98 μm spot size, and a 90° mirror was used to collect Raman spectra. Measurements were performed with 10% laser power (30 mW), with 1 s exposure time and 20 accumulations. As previously demonstarted, the conditions are beneign enough to prevent any laser induced electrolyte decomposition and heating.[1]

As described in Figure S2 an optimum focal spot location was chosen to measure both the sapphire reference peak and the electrolyte. One line scan took around 20 minutes and prior to every new line scan, a PEIS scan with voltage amplitude of 100 mV and frequencies from 100 kHz to 1 Hz, was performed. The large voltage amplitude was chosen for a reasonable signal-to-noise ratio. Linearity was still maintained. After the four hours the interphase impedance has stabilized and a constant areal current of 300uA/cm2 was drawn for ten hours for concentration gradient to form. During polarization, 1D line scans and EIS were continously collected. A laser power of 10% was deemed optimal as it provided good spectral resolution, whilst showing no evidence of thermal heating or oxidation of the solvent. This has been documented elsewhere where no change in the solvent peaks, nor emergence of additional peaks was detected thus there was no evidence of decomposition products. [1] Also, there was no decrease in the overpotential with time, indicating no thermal heating.

Data Processing and Property Calculations

Data processing and analysis was automized as much as possible using an inhouse developed python package pubilcily avaiable on Github. [2]

The Raman spectra were baseline corrected and normalized by the sapphire reference peak as described in the maintext. The normalized spectra were sorted into OCV (first four hours) and galvanotsatic spectra (subsequent ten hours). OCV spectra were used to create the operando calibration line and extract concentrations from the integrated S-N-S bending peak of $[FSI]^-$ as described in the main text. The OCV concentrations were inspected prior to analysis of every cell to verify no unexpected deviation of $c_{s,ini}$. A signal integration heatmap as exemplariyl shown in Figure S8a was created for all cells to determine when filament nucleation started and to detect possible outliers which were disregarded from the subsequent analysis. The time and space resolved concentrations were fit to the following solution of the diffusion equation, derived from concentrated solution theory as described in more detail elsewhere. [2-4]

$$c_s(z,t) = c_s^* + a\left\{\left(\frac{b}{\pi^{0.5}}\right)e^{\left(-\frac{z}{b}\right)^2} - z \cdot erfc\left(\frac{z}{b}\right) - \left(\frac{b}{\pi^{0.5}}\right)e^{\left(-\frac{z+L}{b}\right)^2} + (-z+L)\cdot erfc\left(-\frac{z+L}{b}\right)\right\}$$

with

$$a = \frac{J(1-t_+^0)}{nFD_{app}}\left(1-\frac{\partial lnc_0}{\partial lnc_s}\right)^{-1}$$

$$b = 2\left(D_{app}t\right)^{\frac{1}{2}}$$

According to CST, and under the assumption that transport properties are constant within the ORGA concentration range, the concentration profiles exhibit rotational symmetry, and thus the data points close to the plating side (the dotted line in Figure S3c shows the cut-off at $Z_{normalized}$ = 0.75) can be neglected in the new fitting method. The resulting fit shows an excellent agreement with the original solvent-normalization fitting (see Figure S3d).

Calculation of the transport properties are thoroughly described in Fawdon et al. [2] In short, D_{app} was calculated by linear fitting b (the diffusion length L_D) as a function of the square root of polarization time (see Figure S6a and b). τ_0^+ was calculated from the interfacial gradient a (see Figure S6c and S6d). To calculate χ and κ the electrochemical data was required. PEIS was fit to a simple R_0-($R_{interphase}$-CPE1) circuit using impedance.py. [5] The concentration over potential η_{conc} was calculated by $\eta_{conc} = \eta_{total} - I(R_0 + R_{interphase})$, where η_{total} is measured from the chronopotentiometry data. χ was then determind by a linear for of the weighted gradient according to

$$\chi_M = \frac{F}{2RT(1-t_+^0)} \frac{d\eta_{\text{conc}}}{dln\left(\frac{c_{s,z=L}}{c_{s,z=0}}\right)}$$

as illustrated in Figure S6e and S6f. Lastly, κ was calculated according to

$$\kappa = \frac{\mathrm{L}}{R_0 A}$$

A thorough discussion on the measurment errors and uncertainties can be found in previous publications. [2,4] All properties reported in the main text originate from three separate experiments conducted at each temperature, with the error bars representing the standard deviation of measurements conducted at the same temperature.

Partial Molar Volume and SVF Calculations

The partial molar volumes of the salt V_s and the solvent V_0 were calculated from the density and its dependence on electrolyte concentration. The densities were measured using an Anton Paar DMA 4100 density meter, located in an Argon-filled glovebox. Each measurement was temperature controlled between 20°C and 50°C. The density meter was rinsed with isopropanol (>=99.9%, HPLC grade,Fisher Chemical) at least three times and dried in ambient argon between measurements. The densities are plotted in Figure S1. Partial molar volumes were calculated using the following equations and are plotted in Figure S1.

$$V_s = \frac{M_s - \frac{d\rho}{dc}}{\rho - c\frac{d\rho}{dc}}$$

$$V_0 = \frac{M_0}{\rho - c\frac{d\rho}{dc}}$$

The solvent velocity factors (SVF) required for accurate calculation of τ_0^+ was calculated for each temperature using the following equation and are plotted in Figure S1.

$$SVF = \left(1 - \frac{\partial lnc_0}{\partial lnc_s}\right) = \frac{1}{c_0 V_0}$$

2-Point-Operado Calibration

Before polarization, the ORGA cell is resting under open circuit voltage (OCV) conditions for four hours to enable passivation of the lithium metal electrodes via the formation of a solid electrolyte interphase (SEI). [3] During this resting phase, line scans along the z-axis of the cell are performed every 20 minutes, yielding a total of 12 complete line scans during the cell's resting period. The mean normalized $[FSI]^-$ bending mode area at each z-value (after reaching thermal equilibrium) was calculated. The deviation of individual measurements from their respective mean values is depicted in Figure S3a. An uncertainty of at maximum ±5% around the mean can be observed. Since no systematic offset or drift of the normalized area is detected it can be concluded that a homogeneous lithium concentration equal to the initial electrolyte concentration (= $c_{s,ini}$ = 1 m LiFSI) is measured along the z-axis. As shown in Figure S7 the absolute area of the normalized and integrated $[FSI]^-$ peak is dependent on the z-value (cell height). A similar cell-height dependency trend can be observed for the normalized and integrated G4 peak which indicates the noise originates from surface roughness of the sapphire tube. Consequently, the measurement noise is attributed µm position fluctuations of the here utilized Raman stage. The mean normalized area is the best estimate of $c_{s,ini}$ and thus can serve as a data point for an operando calibration. The origin serves as a second data point enabling the construction of a two-point linear calibration line.

Supporting Figures

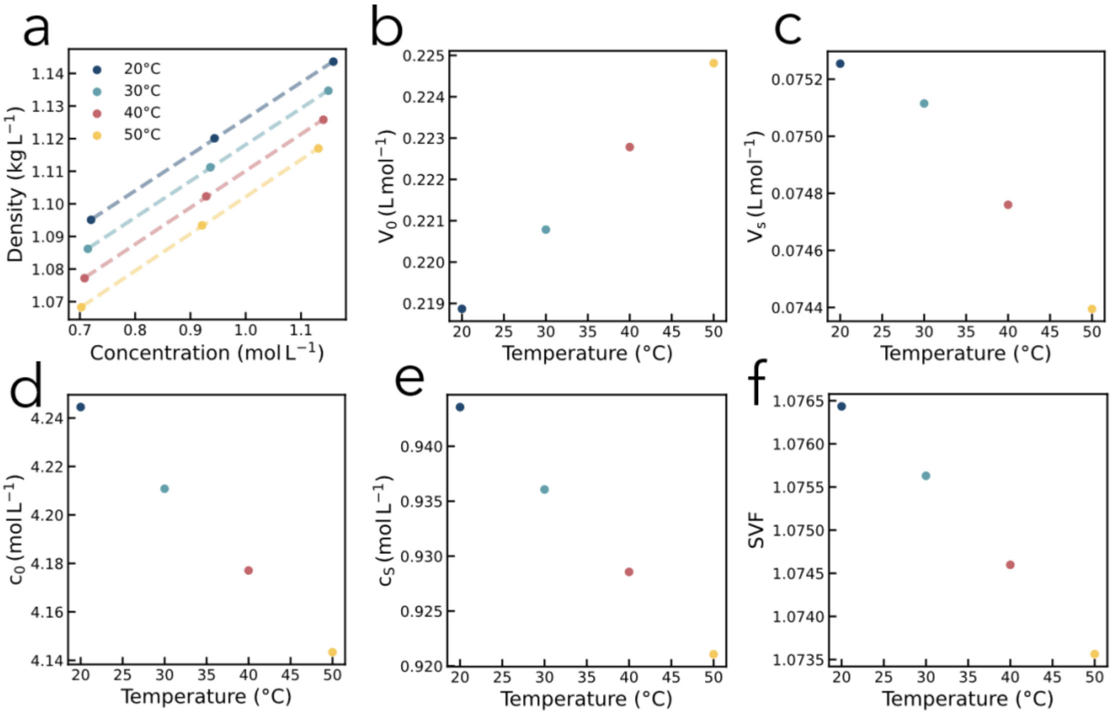

Figure S1: Temperature Resolved: (a) density measurements of LiFSI G4, (b) calculated molar volume (V) of G4 and (c) of LiFSI as well as (d) molar concentration (c_0) of G4, (e) and LiFSI, and (f) calculated SVF.

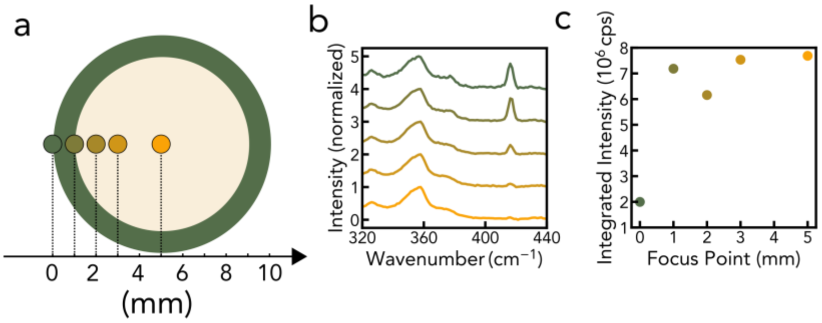

Figure S2: Focal Point Adjustment (a) Schematic of the laser focal point in the ORGA setup. Green indicates sapphire glass and yellow the electrolyte. (b) Relative peak intensity of sapphire and electrolyte when adjust the focal point. The color code corresponds to the same color code in a, and b, (c) Integrated intensity of Raman spectra when focusing on different focal points.

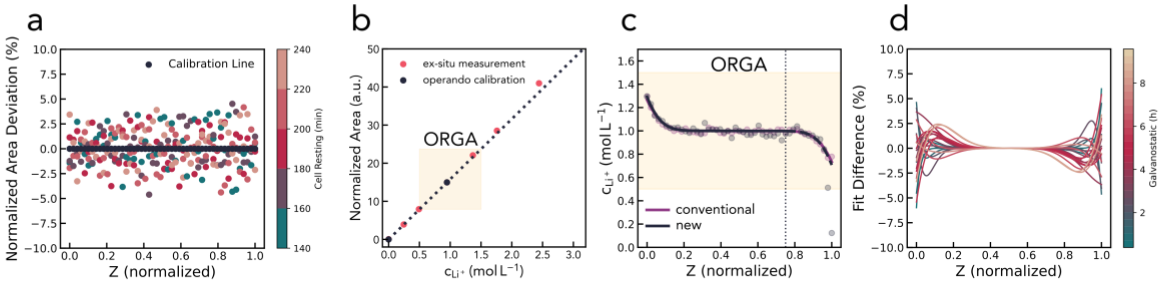

Figure S3: External Reference for normalization and operando calibration. a) shows the mean normalized $[FSI]^-$ bend mode area at each z-value and the respective scattering around it of individual measurements. All areas were collected during OCV conditions. b) Normalized areas as a function of lithium concentration obtained from a conventional calibration line measurement ('ex-situ measurement') is shown in red. The dotted line is a linear fit through the 1M area and the origin ('operando-calibration'). The yellow box indicates the concentration range where ORGA is operated. c) Shows the concentration profiles and their respective fits obtained through the conventional method (solvent normalization and ex-situ calibration) and new method (external reference normalization and operando calibration). The new fitting method neglects all concentration points above Z (normalized) = 0.75 to account for artefacts induced by lithium filaments. d) shows the fit differences residual of the conventional and new processing method.

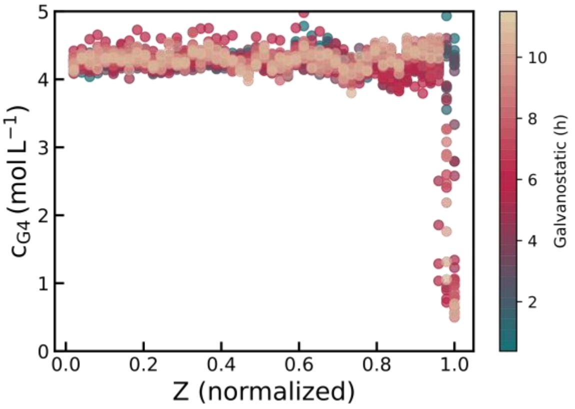

Figure S4: Shown is the concentration profile of G4 after 4 hours of galvanostatic cell operation. Visible is an apparent drop in G4 concentration close to the lithium plating side.

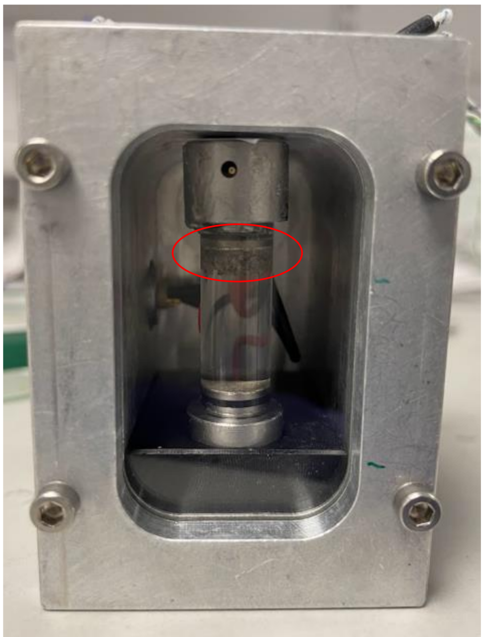

Figure S5: Photograph of the ORGA cell, post- cell operation, revealing the accumulation of dead/mossy lithium at the lithium plating side (red).

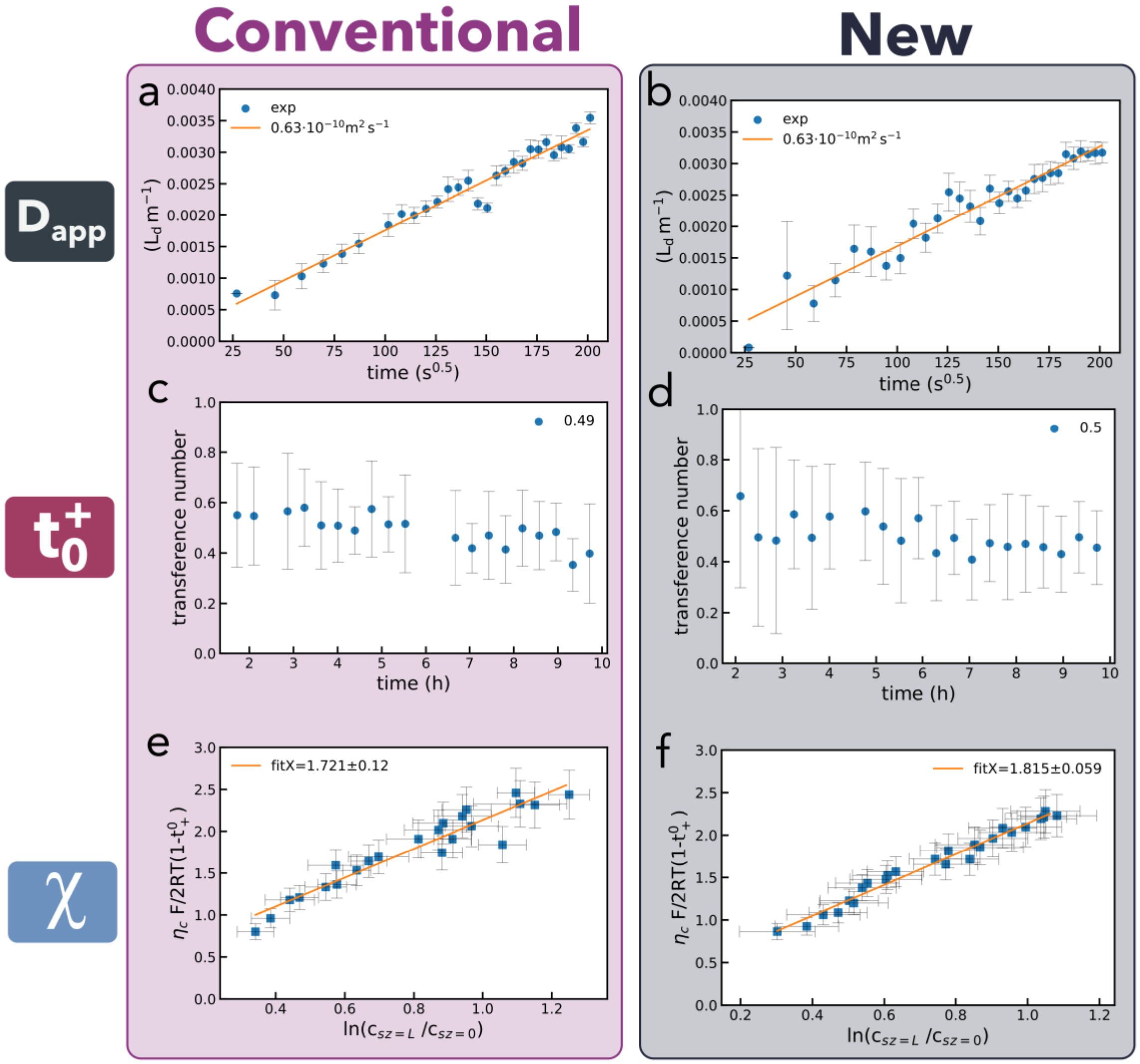

Figure S6: Comparison of electrolyte transport properties (D_{app}, t_0, χ) calculated using the updated ORGA method, i.e. using the internal reference for spectrum normalization and operando calibration (a,c,e), and the previous method using solvent normalization and an ex-situ calibration data set (b,d,f). Shown are D_{app} (a,b) which was calculated by linear fitting the diffusion length L_D as a function of the square root of polarization time, t_0^+ which was calculated from the interfacial gradient a, and χ where electrochemical data was required as described in Data Processing above. No significant differences can be observed using either method.

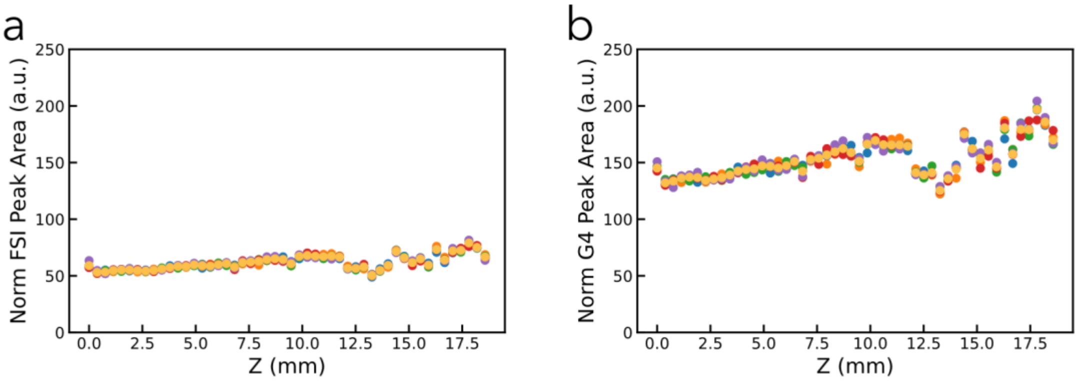

Figure S7: Absolute peak areas as a function of cell height of a) FSI and b) G4. Different colours illustrate different times for each of each line scan.

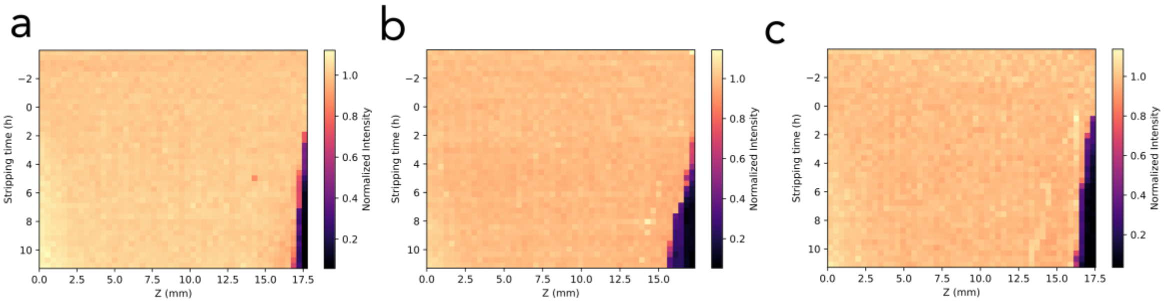

Figure S8: Three additional signal intensity heatmaps collected during ORGA at 30°C.

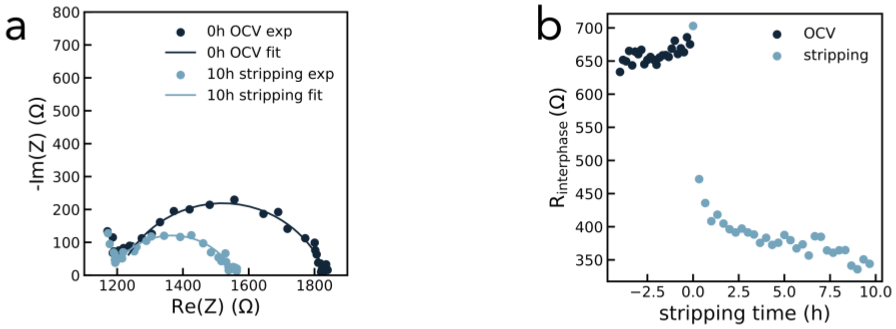

Figure S9: (a) Impedance data and corresponding fit using a circuit described above of an ORGA cell right after assembly (0h OCV) and after 10h of stripping. (b) $R_{interphase}$ as a function of stripping time. A significant impedance drop can be observed which indicates an increase in surface area because of lithium filament nucleation.

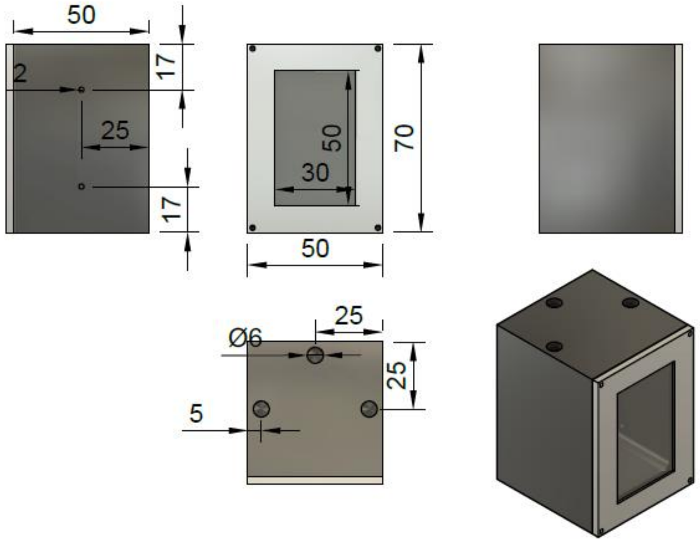

Figure S10: CAD drawing of the temperature chamber designed for the Raman cell. The environmental chamber is heated externally using resistive heaters in the temperature chamber walls.

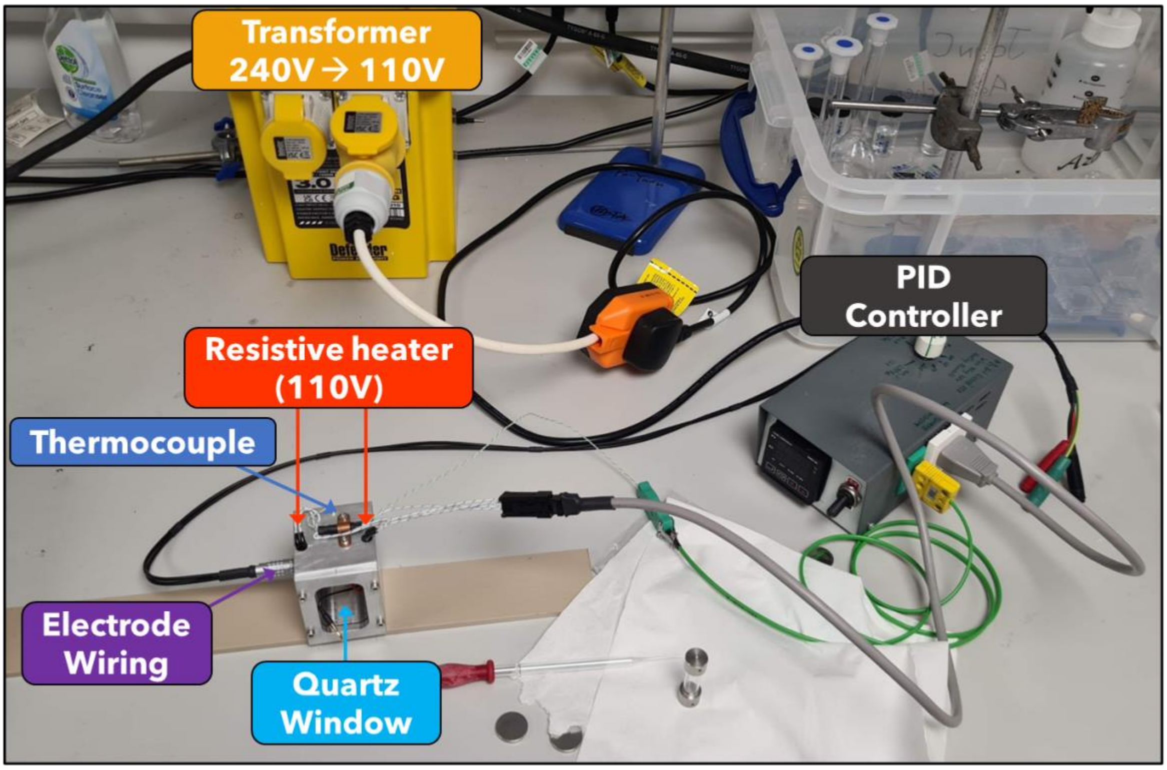

Figure S11: Overview of the individual parts of the ORGA setup with the incorporated thermal chamber.

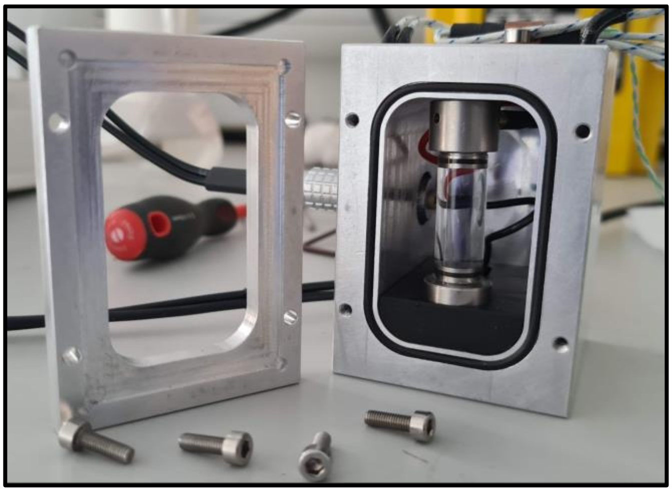

Figure S12: Photograph of the thermal chamber.

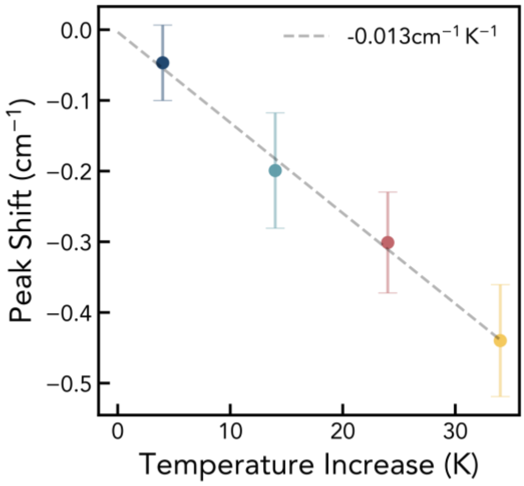

Figure S13: Shows the relative peak shift of the A_{1g} vibrational mode of sapphire as a function of temperature averaged over five points across the cell length over three cells for each temperature respectively. The relative peak shift is determined as the peak position difference of the very first measurement (no heating applied yet) and the one after two hours where thermal equilibrium has been reached.

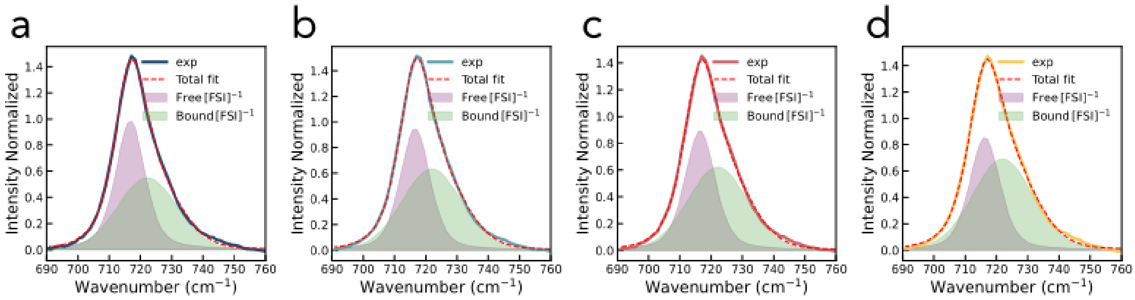

Figure S14: Double pseudo-Voigt fit of the S-N-S bending mode of $[FSI]^-$ as a function of temperature. (a) measured at 20°C and corresponding peak areas of $[FSI]^-_{free}$ = 13.3 and $[FSI]^-_{bound}$=13.1(b) at 30°C and corresponding peak areas of $[FSI]^-_{free}$ = 12.7 and $[FSI]^-_{bound}$= 15.3 (c) at 40°C and corresponding peak areas of $[FSI]^-_{free}$ = 12.3 and $[FSI]^-_{bound}$=15.0 and (d) at 50°C and corresponding peak areas of $[FSI]^-_{free}$ = 11.7 and $[FSI]^-_{bound}$= 17.0

References

[1] J. Fawdon, J. Ihli, F. L. Mantia, and M. Pasta, "Characterising lithium-ion electrolytes via operando Raman microspectroscopy.", Nature Communications 12 (2021), 10.1038/s41467-021-24297-0.

[2] Github: auto_echem, https://github.com/lfo96/auto_echem, last tested on 24.02.2024

[3] B. Jagger and M. Pasta. "Solid electrolyte interphases in lithium metal batteries." Joule 7.10 (2023): 2228-2244.

[3] J. S. Newman and N. P. Balsara, Electrochemical Systems - The Electrochemical Society Series, 4th ed. (Wiley,2021).

[4] J. Zhao, B. Jagger, L. F. Olbrich, J. Ihli, S. Dhir, M. Zysk, X. Ma, and M. Pasta, "Transport and Thermodynamic Properties of KFSI in TEP by Operando Raman Gradient Analysis." ACS Energy Letters 9.4 (2024): 1537-1544.

[5] M. D. Murbach, et al. "impedance. py: A Python package for electrochemical impedance analysis." *Journal of Open Source Software* 5.52 (2020): 2349.
